# Generation and collective interaction of giant magnetic dipoles in laser cluster plasma

**DOI:** 10.1038/s41598-021-95465-x

**Published:** 2021-08-05

**Authors:** A. Andreev, K. Platonov, Zs. Lécz, N. Hafz

**Affiliations:** 1 ELI-ALPS, ELI-HU Nonprofit Ltd., Szeged, Hungary; 2 Max Born Institute (MBI) for Nonlinear Optics, Berlin, Germany; 3 SPb State University, St. Petersburg, Russia; 4 SPb Technical University, St. Petersburg, Russia; 5 Department of Plasma and Nuclear Fusion, Nuclear Research Center, Egyptian Atomic Energy Authority, Abu-Zabal, Egypt; 6 Shanghai Institute of Optics and Fine Mechanics, Shanghai, China

**Keywords:** Laser-produced plasmas, Nanoparticles, Magnetic properties and materials

## Abstract

Interaction of circularly polarized laser pulses with spherical nano-droplets generates nanometer-size magnets with lifetime on the order of hundreds of femtoseconds. Such magnetic dipoles are close enough in a cluster target and magnetic interaction takes place. We investigate such system of several magnetic dipoles and describe their rotation in the framework of Lagrangian formalism. The semi-analytical results are compared to particle-in-cell simulations, which confirm the theoretically obtained terrahertz frequency of the dipole oscillation.

## Introduction

**Figure 1:**
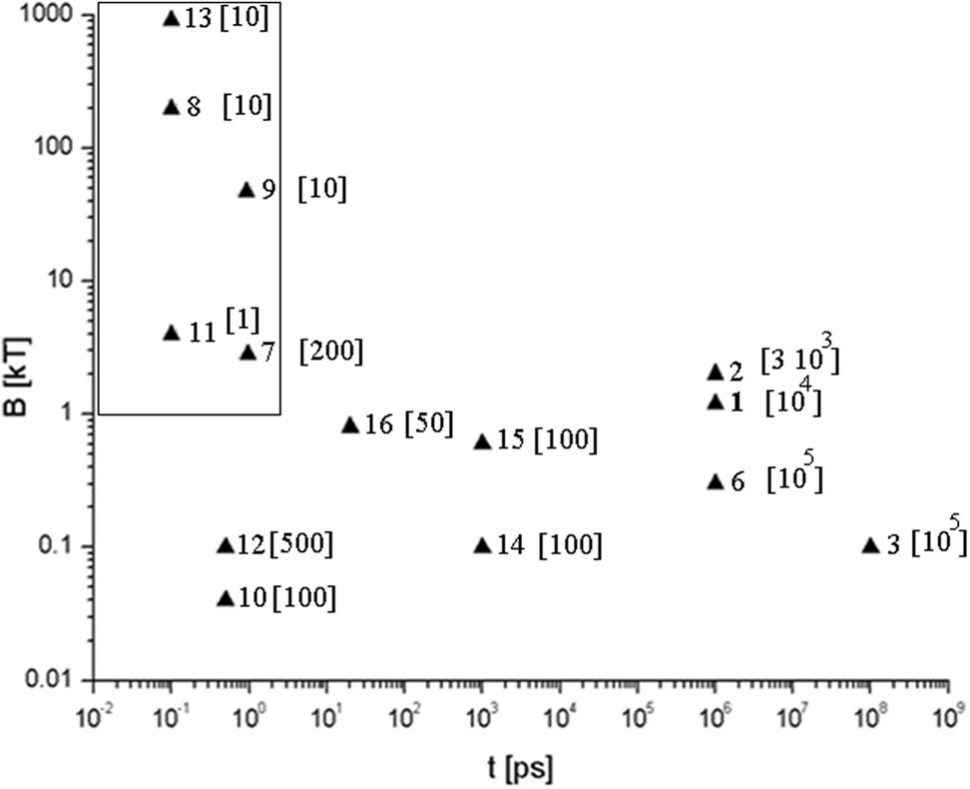
Maximum amplitude of magnetic fields (the number of the triangle is the number in the reference list) its characteristic spatial distribution (in brackets, in μm) and lifetimes obtained recently in real and numerical experiments. The rectangular area corresponds to the range of parameters of the magnetic field generated by the laser cluster plasma studied in our work.

Intense magnetic-field amplitude up to (sub)kilo-tesla has been developed (see Fig. 1) in conventional devices, as superconductive magnets^1–3^, for applied physics, fundamental particle physics and astrophysics^4^. Higher magnetic fields were achieved in Z-pinch experiments^5^ and destructive devices^6^. Recently, owing to the development of high-power lasers, new Z-pinching methods have been investigated in nanowire array targets, which could provide even higher magnetic field amplitudes with micrometer scale lengths^7,8^. Big azimuthal magnetic fields are relatively easy to produce by intense laser pulses on a flat target surface^9–12^. Longitudinal magnetic fields are commonly produced by circularly polarized laser pulses via the effect of inverse Faraday rotation^13,14^. In underdense plasma spatially shaped laser pulses with screw-shaped intensity distribution have been proposed for the generation of near MT axial magnetic field^15^. On the macroscopic level, nanosecond-long pulses with kJ energy have been applied to generate kT magnetic field with a capacitor-coil configuration^16,17^ in a submillimeter spatial domain.

From Fig. 1 one can conclude that for the generation of maximum magnetic field it is better to investigate the case with a relatively short driver pulse and therefore magnetic field lifetime. Such short and strong fields can be interesting in basic science and different applications (see for example^18^).

In the previous publications^19–21^, we proposed a method for generating large-amplitude magnetic-dipole moment based on the electron inertia in cluster rare-gas targets irradiated by circularly polarized ultrashort laser pulses. This magnetic field is stable and remains nearly constant over the timescale of modern short laser pulses (femtosecond durations). In contrast with the uniform density of gaseous underdense plasma, in our method, magnetic dipoles are well-localized at the positions of the overdense clusters (with sizes > 30 nm), and their number equals the number of clusters (~ 10^5^) inside the laser focal volume. A unique feature of this nanoscale magnet is the toroidal current surrounding the cluster. The magnetic field (~ kT) decreases slowly after the laser-cluster interaction, due to the expansion of the ion core, and the decay rate is proportional to the laser electric field and inversely proportional to the cluster mass. It is worth mentioning that in this scheme laser pulses with ∼ mJ energy are sufficient, therefore, magnetization of a material at kHz repetition rate is possible, thanks to the currently available multi-mJ kHz laser systems^22^. The interaction of such clusters through its common magnetic field is similar to that occurring in the area of “magnonics”, which is an emerging field of modern magnetism, and is attracting more and more researchers from various sub-fields of magnetism, materials science and beyond^4^. In this paper we consider different spatial configurations of strong magnetic fields (see Fig. 1) of laser-cluster plasma and investigate its dynamics.

## Analytical model of magnetic moment dynamics in cluster laser plasma

### Cluster dynamics

Let us consider the case when a circularly polarized laser pulse with a vector-potential and electric field $\vec{E}_{L} = - \partial \vec{A}_{L} /\partial ct$, of duration $\tau_{L}$, wavelength $\lambda$, (frequency $\omega$) and intensity $I = cE_{L}^{2} /4\pi$ irradiates the clusters. We suppose that clusters during interaction were partly ionized and their electrons as a spherical shell (charge $- Q = eN_{e}$) surround the cluster ion cores (radius $R_{0}$ and charge $Q$) and are rotated by the circularly polarized laser pulse and the cluster’s own electric fields. The characteristic radius $p$ of the cluster’s electron shell is determined as the distance between the center of the cluster and the point where electron density equals the critical value: $n_{e} (p) = n_{cr}$. This radius determines the square of the absorption spot, the laser energy absorbed by the cluster ${\rm E}_{abs} = \eta I\pi p^{2} \tau_{L}$ and the impulse momentum: $J_{abs} = {\rm E}_{abs} /\omega$, where the absorption coefficient is $\eta \approx 0.1 \div 0.5$^4^. It is worth noting that $p > R_{0}$ because the initial electron density is *n*_*e*_ $\sim 100\;n_{cr}$. Therefore, even if all electrons were heated and their cloud expanded, we would get $p \le R_{0} (n_{e} /n_{cr} )^{1/3} \approx 4.6\;R_{0}$ $1 < p/R_{0} < 4.6$. In general, $p = p(R_{0} ,I)$, when not all electrons are removed, but at an increased laser intensity $p \to 4.6R_{0}$. The electron shell thickness is $p - R_{0} \approx p$, for clusters of small radii or high laser intensities. The mechanical moment of a cluster $J_{abs} = \eta I\pi p^{2} \tau_{L} /\omega$ and the electron mechanical moment $\gamma m_{e} p^{2} \omega \approx \eta I\pi p^{2} \tau_{L} /N_{e} \omega$ give us the number of electrons in the shell:$N_{e} \approx \eta I\pi \tau_{L} /\gamma m_{e} \omega^{2}$ and the total cluster charge $Q = eN_{e}$. The total magnetic moment of a single electron is determined as^5^: $\mu \approx \frac{e}{{8m_{e} \omega \gamma_{L} }}\eta E_{L}^{2} p^{2} \tau_{L}$, where $\gamma_{L} = \sqrt {1 + a^{2} }$ and $a = eE_{L} /m_{e} \omega c$. Outside of cluster: $\vec{H}\left( {\vec{r}} \right) \simeq \mu \left( { - \vec{e}_{x} /\left| {\vec{r}} \right|^{3} + 3x\vec{r}/\left| {\vec{r}} \right|^{5} } \right)$. The maximum value of the cluster’s quasi-stationary magnetic field is estimated as the field on the magnetic dipole axis (x-axis): $H_{\max } = \vec{e}_{x} \vec{H}(p\vec{e}_{x} ) = \frac{2\mu }{{p^{3} }} = \frac{{e\eta E_{L}^{2} \tau_{L} }}{{4m_{e} \omega \gamma_{L} p}},\quad \frac{{H_{\max } }}{{E_{L} }} = \frac{{e\eta E_{L} \tau_{L} }}{{4m_{e} \omega \gamma_{L} p}} = \eta \frac{a}{{\sqrt {1 + a^{2} } }}\frac{{c\tau_{L} }}{4p}$. By comparing it with the numerical results of^2^ we found a good agreement. It must be noted that this formula is correct when $a \le a_{tr} = 2Ze^{2} n_{i} R\lambda /3m_{e} c^{2}$ ($n_{i}$ is initial ion cluster density) and there is no Coulomb explosion in the cluster. The average magnetic field in the laser focal volume with cluster density $n_{cl}$ can be estimated as: $\left\langle H \right\rangle \approx H_{\max } \left( {p/n_{cl}^{ - 1/3} } \right)^{3} = H_{\max } n_{cl} p^{3} ,\;\;n_{cl} p^{3} < 1$ . This field exists during the cluster’s lifetime $\tau_{cl}$ and triggers interaction between the dipoles of many clusters, which we consider below.

The Lagrange function of the shell electrons of the separated “*i*” cluster in the cylindrical coordinate system with the x-axis directed along the circularly polarized laser beam can be written as follows:

1 $$ L = - mc^{2} /\gamma + \frac{e}{c}\frac{{\dot{\vec{r}}}}{c}\left( {\vec{A}\left( {\vec{r}} \right) + \vec{A}_{L} \left( {\vec{r}} \right)} \right) - e\varphi \left( {\vec{r}} \right) + e\mathop \sum \limits_{{\begin{array}{*{20}c} {k = 1,} \\ {i \ne k} \\ \end{array} }}^{{N_{cl} }} \left( {\frac{{\dot{\vec{r}}}}{c}\vec{A}\left( {\vec{r} + \vec{R}_{i} - \vec{R}_{k} } \right) - \varphi \left( {\vec{r} + \vec{R}_{i} - \vec{R}_{k} } \right)} \right) $$

where $\gamma = 1/\sqrt {1 - \beta^{2} } \approx \gamma_{L}$, $\overline{R}_{k}$—is the radius vector of the cluster center of the number «*k*».

Here the scalar potential $\varphi$ of the electrostatic field of the cluster electron shell is determined as $\varphi (r,x) = \frac{Q}{{(r^{2} + x^{2} )^{1/2} }} - \frac{{Q(r^{2} + x^{2} + p^{2} )}}{{2p^{3} }},\;\;R_{0} \le (r^{2} + x^{2} )^{1/2} \le p$; $\vec{A}\left( {\vec{r}} \right)$ is determined as the vector potential of the electron shell (with $Q$ charge and $p - R_{0}$ thickness) rotating around the x-axis with angular velocity of $\dot{\alpha } \approx \omega$ as:

2 $$ \begin{aligned} \vec{A}(r,x) & = - \frac{{Q\dot{\alpha }r}}{2cp}\left( {1 - \frac{{3(r^{2} + x^{2} )}}{{5p^{2} }}} \right)\vec{e}_{\alpha } ,\;\;\;\;R_{0} \le (r^{2} + x^{2} )^{1/2} \le p \hfill \\ \vec{A}(r,x) & = - Q\dot{\alpha }p^{2} r/5c\left( {r^{2} + x^{2} } \right)^{3/2} \vec{e}_{\alpha } ,\;\;\;(r^{2} + x^{2} )^{1/2} \ge p\; \hfill \\ \end{aligned} $$

Outside the cluster, $(r^{2} + x^{2} )^{1/2} > p$, the vector potential of the cluster’s magnetic field coincides with the magnetic dipole potential of the magnetic moment $\mu = Q\dot{\alpha }p^{2} /5c$. The last term of the formula (1) describes the impact of neighboring clusters. We suppose that the electron shells do not touch and do not change their shape. In^3,5^ electron dynamics was investigated without the last term in Eq. (1), therefore our approach is more general.

The distance between any two clusters $\left| {\vec{R}_{ik} } \right| = \left| {\vec{R}_{i} - \vec{R}_{k} } \right| > > \, 2p$ (here $\vec{R}_{i}$ is the radius vector of the center of the *i*-cluster), thus the electrostatic interaction of the nearest clusters is small: ($\varphi \left( {\vec{r} + \vec{R}_{i} - \vec{R}_{k} } \right) \sim \left( {Q/p} \right)exp\left( { - \left| {\vec{r} + \vec{R}_{i} - \vec{R}_{k} } \right|/r_{D} } \right)$ at $\left| {\vec{r} + \vec{R}_{i} - \vec{R}_{k} } \right| > r_{D}$, where $r_{D}$ is the Debye radius of the shell electrons and $r_{D} \approx p$ (if $R_{0} < < \lambda$), $Q < < n_{e} R_{0}^{3}$). From Eq. (2) it follows that $\left| {\vec{A}} \right|\sim \left| {\vec{r}} \right|^{ - 2}$, thus the cluster interaction is mainly magnetic. It is worth noting that in the simulations of many clusters^2^ when the distance between the cluster centres is decreased from 500 to 200 nm (cluster of $R_{0} =$ 50 nm from Xe^+20^), the electrons start moving from one cluster to another, and eventually the electron shells merge. The condition for that follows from^23^ and can be written as: $(\gamma - 1)m_{e} c^{2} > 2eQ/R_{12}$, where $R_{12} \approx n_{cl}^{ - 1/3}$ is the distance between two clusters. Taking into account the finiteness of the electron orbits: $(\gamma - 1)m_{e} c^{2} - eQ/p < 0$, one can rewrite this condition as $R_{12} < 2p$ or $\frac{{1 + a^{2} - \sqrt {1 + a^{2} } }}{{a^{2} }} < \eta c\tau_{L} n_{cl}^{1/3} /2$. The merging of electron clouds is considered in Supplementary for two clusters with different magnetic moments.

Taking into account $2p \ll \left| {\vec{R}_{ik} } \right|\forall i \ne k$, the vector potential of the electrons of the *i*-cluster $\vec{A}\left( {\vec{r} + \vec{R}_{i} - \vec{R}_{k} } \right)$ (with the radius vector $\vec{r}_{i} = \vec{r} + \vec{R}_{i}$) in Eq. (1) can be expanded into a series, and as a result one obtains the following Lagrange function after interaction with laser pulse:

3 $$ L \approx - m_{e} c^{2} /\gamma_{i} + \frac{e}{c}\vec{v}_{i} \vec{A}\left( {\vec{r}} \right) - e\varphi \left( {\vec{r}} \right) + \frac{e}{{2\gamma_{i} m_{e} c}}\left[ {\vec{r} \times \gamma_{i} m_{e} \vec{v}} \right]\mathop \sum \limits_{{\begin{array}{*{20}c} {k = 1,} \\ {i \ne k} \\ \end{array} }}^{{N_{cl} }} \vec{H}\left( {\vec{R}_{ik} } \right) $$

In the cluster’s own fields ~ $\vec{A}$,$\varphi$ it’s the electrons rotate around the x-axis and conserve their momentum. The electrons’ interaction with the nearest clusters (see the last term in Eq. (3)) gives us the following Lagrange equation for the electron momentum:

4 $$ \frac{{d\left[ {\vec{r} \times \gamma_{i} m_{e} \vec{v}_{i} } \right]}}{dt} = \frac{e}{{2\gamma_{i} m_{e} c}}\mathop \sum \limits_{{\begin{array}{*{20}c} {k = 1,} \\ {i \ne k} \\ \end{array} }}^{{N_{cl} }} \left[ {\overline{r} \times \gamma_{i} m_{e} \overline{v}_{i} } \right] \times \overline{H}\left( {\overline{R}_{ik} } \right) $$

By multiplying Eq. (4) with the number of cluster electrons $N_{e}$ and $e/2\gamma_{i} m_{e} c$, one can get the equation of motion of the total magnetic momentum of the cluster: $\vec{\mu }_{i} = eN_{e} \left[ {\vec{r} \times \vec{p}_{i} } \right]/2\gamma_{i} m_{e} c$, where $\gamma_{i} \approx \gamma_{L}$ and $\left| {\vec{\mu }_{i} } \right| \approx \mu$. Its directionality changes in respect to the x-axis. Let us introduce the unit vector $\vec{n}_{i} \left( t \right)$ ($\vec{\mu }_{i} \left( t \right) = \vec{n}_{i} \left( t \right)\mu$) in the direction of the magnetic moment of the $i$-cluster. The magnetic field of the cluster system $\vec{H}\left( {\vec{R}_{ik} } \right)$ outside the cluster ion core is the sum of the fields of the separated dipoles, thus the equation of motion of the separated dipole “*i*” following from (4) is:

5 $$ \frac{{d\vec{n}_{i} (t)}}{dt} = \frac{{eH_{\max } p^{3} }}{{4\gamma m_{e} c}}\sum\limits_{\begin{subarray}{l} k = 1 \\ k \ne i \end{subarray} }^{{N_{cl} }} {\left( { - \frac{{\vec{n}_{i} \times \vec{n}_{k} }}{{R_{ik}^{3} }} + \frac{{3\vec{n}_{i} \times \vec{e}_{ik} (\vec{n}_{k} \vec{e}_{ik} )}}{{R_{ik}^{3} }}} \right)} - \frac{{\vec{n}_{i} (0)\tau_{cl} }}{{(t + \tau_{cl} )^{2} }},\quad \vec{e}_{ki} = \vec{R}_{ki} /R_{ki} $$

The last term added to the right side of Eq. (5) describes relaxation of the cluster’s magnetic momentum due to cluster expansion. This term was obtained by assuming that the cluster fields’ have azimuthal symmetry and $J_{abs}$ is conserved. The latter is considered as an adiabatic invariant when $p$ slowly changes. But the cluster’s magnetic moment $\mu_{i}$ depends on time because electron energy changes $\mu_{i} (t) = eJ_{abs} /2\gamma_{i} (t)m_{e} c$ during the expansion of the cluster. The magnetic field of the rotating and expanding electron cloud ($p > > R_{0}$) is determined by the magnetic moment in a unit volume in which the electron density decreases during expansion. Consequently, one can write the following dependence of the maximum magnetic field on time after the laser pulse leaves the cluster: $H_{\max } (t) \approx H_{\max } \frac{{n_{e} (t)\gamma_{L} }}{{n_{e} (\tau_{L} )\gamma (t)}},\;\;t > \tau_{L}$, where $n_{e} (\tau_{L} ) \approx N_{e} /(4\pi /3)p^{3}$, $p(t) = p + c_{s} t$, and the characteristic velocity $c_{s}$ of the cluster ion (with *A* atomic weight and *Z* charge) was estimated to be $c_{s} \approx \sqrt {Zm_{e} c^{2} (\gamma_{L} - 1)/Am_{p} }$, then $n_{e} (t)/n_{e} (\tau_{L} ) = (p/p(t))^{3}$ and in the case of adiabatic expansion $(\gamma (t) - 1)/(\gamma_{L} - 1) = (p/p(t))^{2}$. From these estimations one can get $H_{\max } (t) = H_{\max } /(1 + c_{s} t/p)$, taking into account electron relativism during expansion. The ratio $p/c_{s} \approx \tau_{cl}$ is the estimation of cluster lifetime.

Therefore, the above estimations of the magnetic moment and cluster magnetic field are correct at $\tau_{L} < \tau_{cl}$. The derivative $d(H_{\max } (t)/H_{\max } )/dt = - \tau_{cl} /(t + \tau_{cl} )^{2}$, as a relaxation term, was added in Eq. (5). It must be noted that at $t < \tau_{cl}$ there is no significant difference between the power and exponential functions of the field, which describes its dependence on time: $H_{\max } (t) = H_{\max } /(1 + c_{s} t/p) \approx H_{\max } (1 - c_{s} t/p);\;\;H_{\max } (t) = H_{\max } \exp ( - c_{s} t/p) \approx H_{\max } (1 - c_{s} t/p)$, and the relaxation term in (5) can be written as $- \vec{n}_{i} /\tau_{cl}$. The simulations have shown that clusters of small radii (tens of nm) demonstrate power law ($\sim t^{ - 1}$), but for larger clusters the exponential time dependence is more appropriate ($\sim \exp ( - c_{s} t/p)$). The analytical and numerical solutions of system (5) for two clusters can be found in Supplementary.

### Magnonic modes of the oscillations of dipole magnetic moments of laser-cluster plasma

Despite the arbitrary position of dipoles in the focal volume, the characteristic distance between the next dipoles $R_{i\;i + 1} \approx n_{cl}^{ - 1/3}$ is determined precisely enough, therefore, the characteristic rotation frequency of the dipoles in (5) is determined accurately as:

6 $$ \Omega \approx eH_{\max } p^{3} n_{cl} /4\gamma m_{e} c,\;\;\;\;\frac{\Omega }{\omega } \approx \frac{{\eta a^{2} c\tau_{L} }}{{16p(1 + a^{2} )}}(p^{3} n_{cl} ) $$

The above equation gives the electron Larmor frequency in the average magnetic field: $< H > = H_{\max } n_{cl} p^{3}$. It is worth noting that at the rotation and oscillation of the magnetic moment is only related to the motion of the electron shell. The period of the dipole turn in Eq. (6) is $\Delta t \approx \Omega^{ - 1}$, thus the condition of the rotation of dipoles during cluster lifetime is $\Omega \tau_{cl} > 1$ or

7 $$ \frac{{\eta a^{2} }}{{16(1 + a^{2} )}}\frac{{\omega \tau_{L} p^{3} }}{{R_{12}{^{3}} \sqrt {(\sqrt {1 + a^{2} } - 1)} }}\sqrt {\frac{{Am_{p} }}{{Zm_{e} }}} > 1 $$

To produce magnonic oscillations (waves) for clusters in laser focal volume, besides the condition described in Eq. (7), the following additional three circumstances must also be fulfilled:

$$ R_{i,i + 1} > 2p\;\;\left( {\text{no cloud touch}} \right);\;\;\;\tau_{L} < p/c\sqrt {Zm_{e} (\sqrt {1 + a^{2} } - 1)/Am_{p} } \;\;\left( {\text{no big expansion}} \right); $$

$$ a < a_{tr} = 2Ze^{2} n_{i} R\lambda /3m_{e} c^{2} \;\;\left( {\text{no Coulomb explosion}} \right). $$

From the above, one can construct the following interval of the possible pulse durations:

$$ \frac{{16(1 + a^{2} )}}{{\eta a^{2} }}\frac{{R_{12}^{3} \sqrt {(\sqrt {1 + a^{2} } - 1)} }}{{\omega p^{3} }}\sqrt {\frac{{Zm_{e} }}{{Am_{p} }}} < \tau_{L} < p/c\sqrt {\frac{{Zm_{e} }}{{Am_{p} }}} \sqrt {(\sqrt {1 + a^{2} } - 1)} . $$

For $a > 1$ the double inequality can be fulfilled if $\frac{8}{\pi \eta }\frac{{a\lambda R_{12}^{3} }}{{p^{4} }}\frac{{Zm_{e} }}{{Am_{p} }} < 1$ or taking into account $R_{12} \ge 2p$ it can be written as $\frac{p}{\lambda } > a\frac{64}{{\pi \eta }}\frac{{Zm_{e} }}{{Am_{p} }}$. Because $p \approx 4R_{0}$ one has $\frac{{R_{0\;} }}{\lambda } > a\frac{16}{{\pi \eta }}\frac{{Zm_{e} }}{{Am_{p} }}$ or $a < \frac{333A\eta }{Z}\frac{{R_{0\;} }}{\lambda }$. The absence of Coulomb explosion limits vector potential: $a < \frac{{2\pi Zn_{i} }}{{3n_{cr} }}\frac{{R_{0} }}{\lambda } \approx 130Z\frac{{R_{0} }}{\lambda }$ ($n_{i} /n_{cr} \approx 60$), but for heavy clusters (for example Xe^+20^, Au^+30^) it fulfills automatically at the conditions of the double inequality. At the maximal $a = \frac{333A\eta }{Z}\frac{{R_{0\;} }}{\lambda }$ both limits of inequality coincide and determine laser pulse duration as: $\frac{{c\tau_{L} }}{\lambda } = N_{L} \approx \frac{30}{\pi }\sqrt {\frac{{R_{0\;} }}{\eta \lambda }}$ thus it is only one point on the plane $(c\tau_{L} /\lambda ;a)$. If now laser field amplitude decreases $a < \frac{333A\eta }{Z}\frac{{R_{0\;} }}{\lambda }$, then laser pulse duration falls in the following interval of the double inequality:

8 $$ \frac{0.5}{\eta }\sqrt{\frac{Z}{A}}  \sqrt a < \frac{{c\tau_{L} }}{\lambda } < \frac{{170R_{0} }}{\lambda \sqrt a }\sqrt{\frac{A}{Z}}  $$

From (8), one can draw the area in the plane $(c\tau_{L} /\lambda ,\;a)\;\;$, where the effect of dipole rotation is realized:

If $\eta ,\;Z$ are the functions of $a,\;\tau_{L} ,\;R_{0}$, then the shapes of the blue and red lines will change, but the shape of the curvilinear “triangle” will be similar. It is true at maximum cluster density, when $R_{12} = n_{cl}^{ - 1/3} = 2p = 8R_{0}$, but if density decreases, this “triangle” shifts to the right side and transforms into a “trapezoid” after the Coulomb limit $a_{tr}$ is reached.

At some conditions, Eq. (5) has solution in the form of waves as: $n_{s} (t) \sim \sin (s\chi - \Omega t)$. Let us consider a chain of $N_{cl}$ clusters located on the x-axis at $b$ distance from one another. In the initial state, the dipoles are in a stable equilibrium and the right-hand sides of Eq. (5) are equal to zero. The direction of a linear chain is parallel to the dipole orientation. Let us consider a small perturbation of magnetic moment of the $s$-th cluster in a chain:$\vec{n}_{s} \left( t \right) = \vec{e}_{x} + \delta \vec{n}_{s} \left( t \right),t \le \tau_{cl}$. Such a variant can be realized if the angle between laser beam and cluster flux is small. System (5), in this case, transforms into the linear system of the ordinary differential equations:

9 $$ \frac{{d\delta \vec{n}_{s} \left( t \right)}}{dt} = \tilde{\Omega }\left( \begin{gathered} - 2\delta \vec{n}_{s} + \delta \vec{n}_{s - 1} + \delta \vec{n}_{s + 1} + \frac{1}{8}\left( { - 2\delta \vec{n}_{s} + \delta \vec{n}_{s - 2} + \delta \vec{n}_{s + 2} } \right)  \hfill \\ + \frac{1}{27}\left( { - 2\delta \vec{n}_{s} + \delta \vec{n}_{s - 3} + \delta \vec{n}_{s + 3} } \right) + \cdots  \hfill \\ \end{gathered} \right) \times \vec{e}_{x} - \frac{{\delta \vec{n}_{s} }}{{\tau_{cl} }},\;\;\;\;\tilde{\Omega } = \frac{{eH_{max} p^{3} }}{{4\gamma m_{e} cb^{3} }} $$

From (9) it can be seen that the selected cluster mainly interacts with the nearest clusters and $\delta n_{s\;x} (t)\sim \exp ( - t/\tau_{cl} )$. We search for a solution of (9) in the form of a travelling dissipating wave: $\delta \vec{n}_{s} \left( t \right) = \delta \vec{n} \cdot exp\left( {i\left( {\chi /b} \right)sb - i\Omega t - t/\tau_{cl} } \right)$, where $sb$ is the coordinate of the s-th cluster, and $\chi /b$ is the wave vector of magnonic oscillations. For the wave amplitude $\delta \vec{n}$ from (9) one can get the following system of homogeneous equations:

10 $$ \begin{aligned} - i(\Omega + i\tau_{cl}^{ - 1} )\delta \vec{n} & = - 4\tilde{\Omega }\left( {\sin^{2} (\chi /2) + \frac{1}{8}\sin^{2} (\chi ) + \frac{1}{27}\sin^{2} (3\chi /2) + \cdots } \right)\delta \vec{n} \times \vec{e}_{x} \hfill \\ \delta \vec{n} & = (0;\delta n_{y} ;\delta n_{z} )\;\;\; \hfill \\ \end{aligned} $$

The determinant of system (10) gives us the dispersion equation of magnonic oscillation

11 $$(\Omega + i\tau_{cl}{^{ - 1}} )^{2} + 16\tilde{\Omega }^{2} (\sin^{2} (\chi /2)  + \frac{1}{8}\sin^{2} (\chi ) + \frac{1}{27}\sin^{2} (3\chi /2) + \cdots)^{2} = 0\;\;\; $$

and the dispersion law of magnonic oscillations:

12 $$ \begin{aligned} \Omega (\chi ) & = 4\tilde{\Omega }(\sin^{2} (\chi /2) + \frac{1}{8}\sin^{2} (\chi ) + \frac{1}{27}\sin^{2} (3\chi /2) + ...) - i\tau_{cl}^{ - 1}  \hfill \\ & = - 2\tilde{\Omega }\int\limits_{0}^{\chi } {(\chi - \xi )\ln (2\sin (\xi /2))d\xi } \; - i\tau_{cl}^{ - 1} \;\; \hfill \\ \end{aligned} $$

At small values of $\chi$ Eq. (12) is quadratic:$\Omega (\chi ) \approx \tilde{\Omega }\chi^{2} (3/2 - \ln (\chi )) - i\tau_{cl}^{ - 1} ,\;\;\chi \to 0$. The magnon can have left or right circular polarization: $- i\delta n_{y} \pm \delta n_{z} = 0$, $\delta \vec{n}_{s} \left( t \right) = \delta n_{y} \left( {\overline{e}_{y} \pm i\overline{e}_{z} } \right)exp\left( {i\chi s - i\Omega t - t/\tau_{cl} } \right)$.

If the boundary conditions are $\delta \overline{n}_{0} (t) = \delta \overline{n}_{{N_{cl} + 1}} (t) = 0$ (fixed clusters at the ends of the chain), then the travelling waves form a standing wave: $\delta \vec{n}_{s} (t) = \delta n_{y} \;\sin (\chi s)(\overline{e}_{y} \cos (\Omega (\chi )t + \psi ) \pm \overline{e}_{z} \sin (\Omega (\chi )t + \psi ))$, and the set of magnonic wave vectors becomes discrete $\;\;\;\chi_{l} = \pi l/(N_{cl} + 1),\;\;l = 1,\;2...N_{cl}$. The general solution in this case is a set of standing magnonic waves with the different wave vectors:

13 $$ \delta \vec{n}_{s} (t) = \sum\limits_{l = 1}^{{N_{cl} }} {\delta n_{ly} \;\sin (\chi_{l} s)(\overline{e}_{y} \cos (\Omega_{l} t + \psi_{l} ) \pm \overline{e}_{z} \sin (\Omega_{l} t + \psi_{l} ))} , $$

where $\Omega_{l} = \Omega (\chi_{l} )$. In the case of the chain from two clusters: $N_{cl} = 2,\;\chi_{1} = \pi /3,\;\chi_{2} = 2\pi /3,\;\Omega_{1} = \Omega_{2} = 3\tilde{\Omega }a$, the solution of Eq. (13), as shown in Supplementary, will be correct for the perturbation of arbitrary amplitude. A change in the boundary conditions, for example, the ring with a big radius, $\overline{n}_{1} \left( t \right) = \delta \overline{n}_{{N_{cl} + 1}} \left( t \right)$ modifies the discrete values of the wave vectors: $\;\;\;\chi_{l} = 2\pi l/N_{cl} ,\;\;l = 0,\;1...N_{cl} - 1$ with the conservation of the form of the solution of Eq. (13).

To demonstrate the effect of the dipole rotation and check analytical model, we provide numerical simulations for the interaction of a laser beam with cluster media using the 3D PIC code in the next section.

## Simulation results of magnetic dipole interaction

### Simulations of the interaction of a laser pulse with several clusters (nano-droplets)

The simulation tool we use is the 3D EPOCH particle-in-cell code^24^. We assume an already-ionized spherical target (cluster) with an initial radius $R_{0}$. First, we consider Xe clusters with an ion charge state of $Z$ = 20 (from tunnel ionization) and a number density of $n_{0}$ = 10^22^ cm^−3^. The corresponding electron density in the target is 2 × 10^23^ cm^−3^, which is equivalent to $n_{e} = \, 182n_{cr}$ for $\lambda_{L}$ = 1 μm laser wavelength. The incoming laser pulse is a circularly polarized plane wave (the simulation domain is smaller than the laser focal spot size) having a Gaussian temporal field profile, $I\left( t \right) \, = \, I_{L} exp\left[ { - \left( {t \, - \, t_{L} } \right)^{2} /t_{L}^{2} } \right]$.The peak intensity of the circularly polarized Gaussian laser pulse is $I_{L}$ = 2 × 10^18^ W/cm^2^ and its duration is varied during the simulations. The simulation domain has a volume of 1.5 × 1 × 1 μm^3^ represented by 600 × 400 × 400 grid cells, and the target plasma is represented by 10 ion and 100 electron macroparticles per cell. At such high resolution, one pseudo-electron contains only ∼10 real electrons and the total number of macroparticles used to represent the target plasma is 10^7^. Since the radius of the electron trajectory quickly exceeds the width of the simulation box, we use absorbing boundaries for electrons and open boundaries for electromagnetic waves. We have tested the periodic boundary conditions in the transverse direction too. The result is approximately the same: the only difference is a small fluctuation in the temporal evolution of the magnetic field, which is attributed to several energetic electrons crossing the simulation box in a transversal plane in the case of the periodic boundary.

The laser pulse duration is 6 fs. The simulations were performed for four $Xe^{ + 20}$ clusters (spheres with a radius of 40 nm). The coordinates of the four droplet (cluster) centres in the simulation domain 1 × 1 × 1.5 μm are as the following: Droplet 1: x = 260 nm, y = − 60 nm, z = 0; Droplet 2: x = 340 nm, y = 60 nm , z = 0; Droplet 3: x = 300 nm, y = 0, z = 60 nm; Droplet 4: x = 300 nm, y = 0, z = − 60 nm. Laser pulse propagates along the x-axis. The cluster location in the simulation box is shown in Fig. 3.

Figure 4 shows the dynamics of the magnetic field components of clusters no. 1 and 4 (simulated maximum values in the cluster centers) and the analytical dependence of the same components on time, as calculated from Eq. (5): $\vec{H}_{i} (t) = H_{\max } \vec{n}_{i} (t)$.

**Figure 2:**
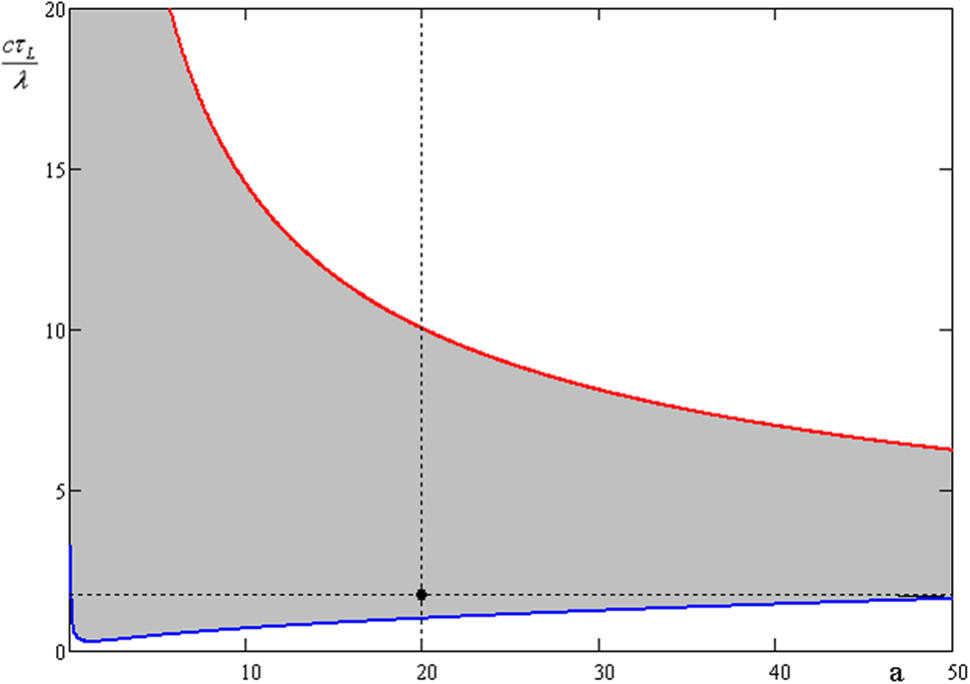
The range (Eq. 8, grey area) of laser parameters where magnonic oscillations of cluster plasma can be realized. Here $Z = 30,\;A = 196,\;\;\eta = 0.2,\;R_{0} = 100\;{\text{nm}},\;\lambda = 1000\;{\text{nm}}$, and $p = 4R_{0}$ . The distance between the nearest clusters is $R_{12} = 2p = 800\;{\text{nm}}$. The black dot shows the laser pulse of 6 fs and 5.6 × 10^20^ W/cm^2^, used in our PIC simulations.

**Figure 3:**
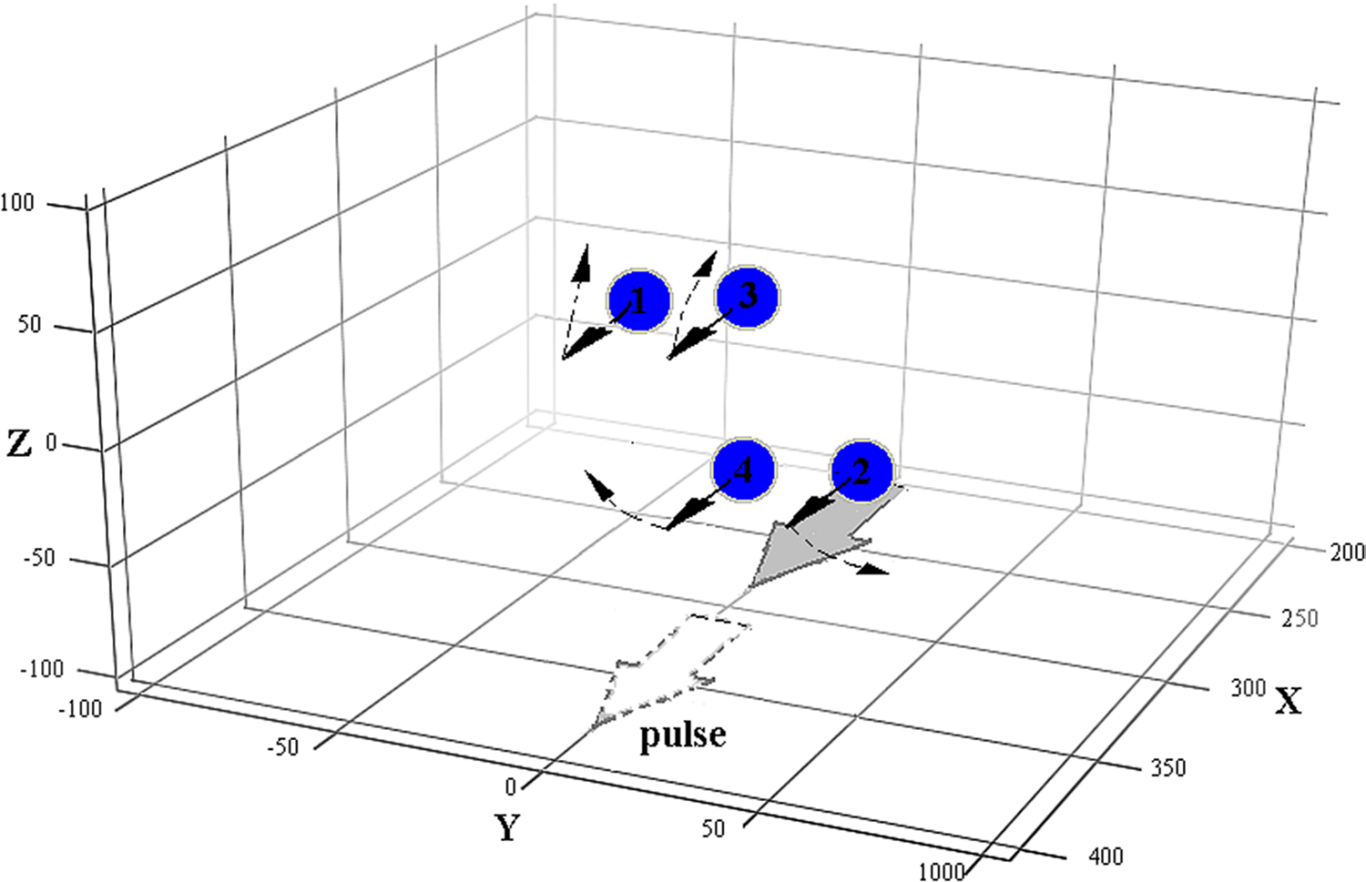
The positions of the four clusters in the simulation box. The X, Y and Z coordinates are given in nanometers. The laser pulse is circularly polarized and propagates along the x-axis. Pulse positions during interaction are shown by gray solid arrow and after by dash arrow. The solid arrows show the directionalities of cluster magnetic moments during laser pulse action. The dash arrows show the directions of magnetic moment rotation because magneto-dipole interaction after finishing of laser pulse.

**Figure 4:**
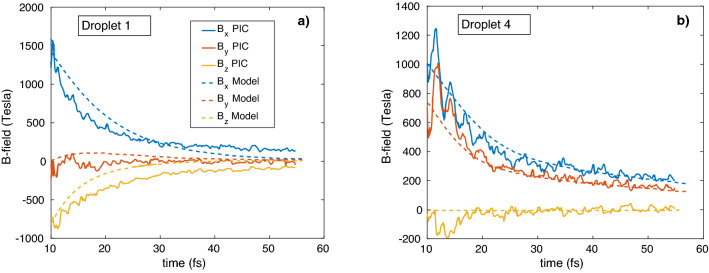
The time dependence of the magnetic field components for the Xe^+20^ cluster irradiated by a laser pulse having an intensity of I_L_ = 2 × 10^18^ W/cm^2^ and 6 fs duration, obtained by PIC simulations (modulated lines) and analytically (smooth lines of the same colours) (**a**) Cluster #1, (**b**) Cluster #4 .

**Figure 5:**
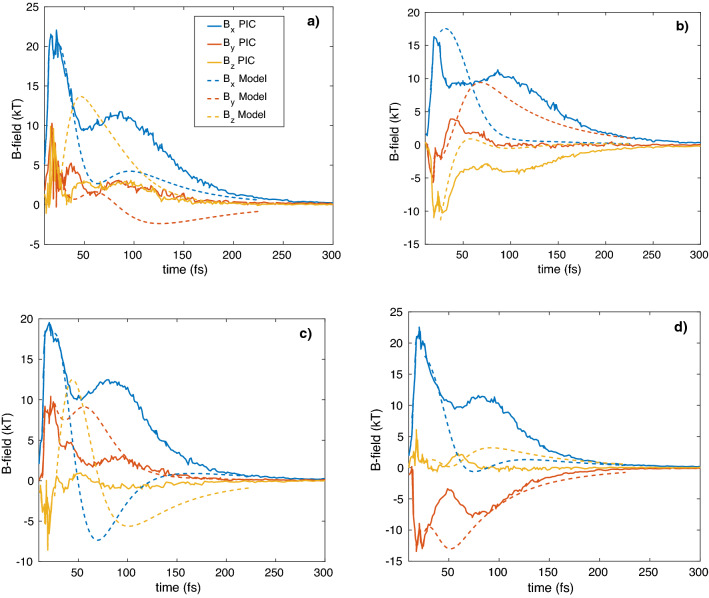
The time dependence of the magnetic field components for Au^+30^ 4-x clusters irradiated by a laser pulse of I_L_ = 5.6 × 10^20^ W/cm^2^ intensity and 6 fs duration, obtained by PIC simulations (modulated lines) and analytically (smooth lines of the same colors).

In contrast with a single cluster^19,21^, when the X component of the magnetic field is higher than the other components, in the system of closely located clusters the magnetic field components are significant in the directions perpendicular to laser the axis, which is caused by the interaction of the dipole electron shells. The relaxation time in Fig. 3 is $\tau_{cl} \approx$ 20 fs, therefore this is the main term in Eq. (5) and the solution is: $\overline{n}_{i} (t) \approx \overline{n}_{i} (0)/(1 + t/\tau_{cl} )\;\;i = 1,2 \ldots 4\;\;$. The initial values of $\vec{n}_{i} \left( 0 \right)$ are taken from the simulations, but if $n_{i\;y,z} (0) = 0,\;n_{i\;x} (0) = 1,\;\;i = 1,2 \ldots 4\;\;$, no transversal (y, z) field components appear during the ~ 60 fs time window. The above simulations do not show magnonic oscillations ($\Omega \tau_{cl} \approx 0.4$), because the simulation parameters do not satisfy the conditions (8) and the parameter set fall outside the characteristic triangle of Fig. 2 for this case. In Fig. 3 such parameters of calculation coexist to appearance and disappearance of black solid arrows, which denote cluster magnetic moments. There is no rotation of magnetic moments (dash arrows in Fig. 3) in this case. To confirm the effect of magnetic dipole rotation (the appearance of dash arrows in Fig. 3 ), we simulated Au^+30^ clusters of 100 nm radius (bigger $\tau_{cl}$) at a higher laser intensity of 5.6 × 10^20^ W/cm^2^ (bigger $\Omega$) and 6 fs pulse duration and a super-Gaussian shape (see black dot in Fig. 2). This time the simulation box was bigger: 3 × 3 × 3 μm^3^. The cluster locations in the box are the following: R_1_ = (510, − 490, 0), R_2_ = (1490, 490, 0), R_3_ = (1000, 0, 400), R_4_ = (1000, 0, − 400) nm. The model calculations used exponential damping and initial conditions taken from the simulations when the laser pulse ends. The model parameters ($p = 4.2R_{0}$) were chosen to show the best agreement between the model and simulation curves of Fig. 4. The results of the simulations and the analytical model show that the evolution of the magnetic field components is not only relaxation, but clear oscillations of the Y and Z components (even during one period) prove magnetic interaction of the clusters. In Fig. 3 by dash arrows are shown the rotation directions of cluster magnetic moments, which coexist to PIC simulation results of Fig. 5.

From Fig. 5 one can conclude that the developed analytical model agrees well with the PIC simulations qualitatively, but the amplitudes and times of the changing field components are different.

To confirm the multi-oscillations of the magnetic field, which in Fig. 5 are damped by relaxation, special simulations were done with immobile ions where the collisionless relaxation time was sufficiently long. Supplementary shows that in this case the magnetic field components oscillate clearly, suggesting the mutual rotation of the cluster’s magnetic moments and the realization of standing waves, i. e. magnons.

Period *T* of the oscillations of the projections of the cluster’s magnetic moments in Supplementary and in Fig. 5 corresponds to the THz range of frequencies. Therefore, the system of clusters radiates secondary transversal electromagnetic waves in the THz range^3^. This secondary THz radiation can be coherent in terms of cluster number if the focal area is shorter than the wavelength of THz radiation. We plan to investigate plural oscillations of the cluster’s magnetic moments with mobile ions by increasing the radii of clusters in a subsequent publication.

## Conclusion

The generation, interaction and dissipation of the giant magnetic moments of clusters in laser- plasma interaction was demonstrated in the focal volume of circularly polarized relativistic intense laser pulses, interacting with the clusters of radii from tens up to hundreds of nanometers. It is shown that at a laser intensity of about 10^20^ W/cm^2^, one can get a magnetic field of up to 0.5 MegaTesla at lifetime of hundreds femtoseconds. The generated super strong magnetic field in a configuration of a magnetic trap can slow down thermal expansion of a thermonuclear target and increase burning time of nuclear fuel. Beside such ultra-strong magnetic field the intense THz oscillations of magnetic moments of clusters (magnons) can be generated inside focal volume at cluster density above 10^11^ cm^−3^. For the first time we determined the dispersion relations of these magnonic waves of cluster plasma. We have shown that the discovered waves appear at specific laser-plasma parameters, partly because its duration must be high enough to convert the absorbed impulse momentum into cluster electron one, but not exceed cluster plasma lifetime, thus the optimum conditions for the realization of these effects are enough large diameter heavy clusters. From the current results, it is anticipated that the rotation of electron cloud around the ion core will result in intense synchrotron emission with small angular spread perpendicular to laser axis. It is worth mentioning that in this scheme circular polarized laser pulses with ∼ mJ energy are sufficient to produce strong magnetic field (~ few kT) decreases slowly after the laser-cluster interaction, therefore, magnetization of a material at kHz repetition rate is possible, thanks to the currently available multi-mJ kHz laser systems. The interaction of such clusters through its common magnetic field is similar to that occurring in the area of “magnonics”, which is an emerging field of modern physics, and is attracting more and more researchers from various sub-fields of magnetism, materials science and beyond.

## Supplementary Information

Supplementary Information.

## References

[1] Nakamura D, Ikeda A, Sawabe H (2018). Record indoor magnetic field of 1200 T generated by electromagnetic flux-compression. Rev. Sci. Instrum..

[2] Hansel S, Müller H (2006). The Humboldt high magnetic field center at Berlin. J. Phys. Conf. Ser..

[3] LANL https://nationalmaglab.org/user-facilities/pulsed-field-facility (2012).

[4] Kruglyak V (2010). Magnonics. J. Phys. D: Appl. Phys..

[5] Remington BA, Drake RP, Ryutov DD (2006). Experimental astrophysics with high power lasers and Z pinches. Rev. Mod. Phys..

[6] Bykov AI (2001). VNIIEF achievements on ultra-high magnetic fields generation. Phys. B.

[7] Kaymak V, Pukhov A, Shlyaptsev VN, Rocca JJ (2016). Nanoscale ultradense Z-pinch formation from laser-irradiated nanowire arrays. Phys. Rev. Lett..

[8] Lécz Z, Andreev A (2018). Laser-induced extreme magnetic field in nanorod targets. New J. Phys..

[9] Sarri G, Macchi A (2012). Dynamics of self-generated, large amplitude magnetic fields following high-intensity laser matter interaction. Phys. Rev. Lett.

[10] Abicht F, Braenzel J, Priebe G (2014). Tracing ultrafast dynamics of strong fields at plasma-vacuum interfaces with longitudinal proton probing. Appl. Phys. Lett..

[11] Abicht F, Bränzel J, Priebe G (2016). Tracing dynamics of laser-induced fields on ultrathin foils using complementary imaging with streak deflectometry. Phys. Rev. AB.

[12] Huang LG, Takabe H, Cowan TE (2019). Maximizing magnetic field generation in high power laser-solid interactions. High Power Laser Sci. Eng..

[13] Najmudin Z, Tatarakis M, Pukhov A (2001). Measurements of the Inverse Faraday Effect from Relativistic Laser Interactions with an Underdense Plasma. Phys. Rev. Lett..

[14] Tatarakis M, Watts I, Beg FN (2002). Measuring huge magnetic fields. Nature.

[15] Lécz Z, Konoplev IV, Seryi A, Andreev A (2016). GigaGauss solenoidal magnetic field inside bubbles excited in under-dense plasma. Sci. Rep..

[16] Daido H, Miki F, Mima K (1986). Generation of a strong magnetic field by an intense CO_2_ laser pulse. Phys. Rev. Lett..

[17] Tikhonchuk V, Bailly-Grandvaux M, Santos J, Poyé A (2017). Quasistationary magnetic field generation with a laser-driven capacitor-coil assembly. Phys. Rev. E.

[18] Du B, Wang X-F (2017). Influence of an external axial magnetic field on betatron radiation from the interaction of a circularly polarized laser with plasma. Phys. Plasmas.

[19] Lecz Zs, Andreev A (2020). Magnetic dipole moment generated in nano-droplets irradiated by circularly polarized laser pulse. Phys. Rev. Res..

[20] Andreev A, Platonov K (2020). Dynamics and emission of relativistic magnetic dipoles of a laser cluster plasma. JETP Lett..

[21] Andreev A, Platonov K (2021). Generation of superstrong quasi-stationary magnetic fields in laser cluster plasma. Quantum Electron..

[22] Toth S (2020). SYLOS lasers—The frontier of few-cycle, multi-TW, kHz lasers for ultrafast applications at extreme light infrastructure attosecond light pulse source. J. Phys. Photonics.

[23] Landau, L. & Lifshiz, E. *Theoretical Physics: Mechanics*, v.1 (1999)

[24] Arber TD, Bennett K, Brady CS, Lawrence-Douglas A, Ramsay MG, Sircombe NJ, Gillies P, Evans RG, Schmitz H, Bell AR, Ridgers CP (2015). Contemporary particle-in-cell approach to laser-plasma modeling. Plasma Phys. Control. Fusion.

