# Supplementary material for: Generation and collective interaction of giant magnetic dipoles in laser cluster plasma
Source: Sci Rep. 2021 Aug 5;11:15971. doi: 10.1038/s41598-021-95465-x (PMC8342715; doi:10.1038/s41598-021-95465-x)
Supplement: Supplementary file 1 — Supplementary Information. [file 41598_2021_95465_MOESM1_ESM.docx]

**Supplementary file**

**Two closely placed clusters**

For two identical dipoles directed in parallel during the initial moment of time, the system (7) has the following analytical solution in the absence of dissipation ($\tau_{cl}\to\infty$):

$\begin{matrix} n_{1\parallel}(t)=n_{2\parallel}(t)=\cos\theta\\ n_{1\perp x}(t)=n_{2\perp x}(t)=\sin\theta\cos(3\Omega t\cos\theta) \\ n_{1\perp y}(t)=n_{2\perp y}(t)=-\sin\theta\sin(3\Omega t\cos\theta) \end{matrix}$, (1S)

where the subscript designate parallel and perpendicular directions relative to the dipole axes, i.e. the components of the magnetic moment; $\theta$ is the angle between the laser pulse wave vector and the dipole axis; the x, y co-ordinates are in a plane perpendicular to the dipole axis. The motion of the magnetic moments is reduced to their precession around the axis with the angular speed: $3\Omega\cos\theta$. In a close cluster arrangement, the electron clouds may overlap..

The system of two clusters motion equations with unequal modules of magnetic moments $\mu_{1,2}$ is as the following:

$\begin{matrix} \frac{\vec{n}_{1}(t)}{dt}=\frac{e\mu_{2}}{2\gamma m_{e}c}\left(-\frac{\vec{n}_{1}\times\vec{n}_{2}}{R_{12}^{3}}+\frac{3\vec{n}_{1}\times\vec{e}_{12}(\vec{n}_{2}\vec{e}_{12})}{R_{12}^{3}}\right)-\frac{\vec{n}_{1}(0)\tau_{cl}}{(t+\tau_{cl})^{2}} \\ \frac{d\vec{n}_{2}(t)}{dt}=\frac{e\mu_{1}}{2\gamma m_{e}c}\left(-\frac{\vec{n}_{2}\times\vec{n}_{1}}{R_{12}^{3}}+\frac{3\vec{n}_{2}\times\vec{e}_{21}(\vec{n}_{1}\vec{e}_{21})}{R_{12}^{3}}\right)-\frac{\vec{n}_{2}(0)\tau_{cl}}{(t+\tau_{cl})^{2}} \end{matrix}$ (2S)

It is visible that unlike in the case of identical clusters in system (2S), there are two frequencies in Eq. system (2S): $\tilde{\Omega}_{2}=\frac{e\mu_{2}}{2\gamma m_{e}cR_{12}^{3}}, \tilde{\Omega}_{1}=\frac{e\mu_{1}}{2\gamma m_{e}cR_{12}^{3}}$.

The system (2S) can be solved only numerically. In the case of immobile ions ($\tilde{\Omega}_{1,2}\tau_{cl}>>1$), dissipation is absent and the dipole moments $\vec{n}_{1,2}(t)$ undergo unlimited oscillations. The solution (2S) allows us to find a magnetic field vector in the center of each cluster $\bar{H}_{1,2}(t)=2\mu_{1,2}\bar{n}_{1,2}(t)/p^{3}$ and outside clusters:

$\vec{H}(\vec{r},t)=\sum_{k=1}^{2} \left(-\frac{\mu_{k}\vec{n}_{k}(t)}{\left|\vec{r}-\vec{r}_{k}\right|^{3}}+\frac{3(\vec{r}-\vec{r}_{k})(\mu_{k}\bar{n}_{k}(t)(\vec{r}-\vec{r}_{k}))}{\left|\vec{r}-\vec{r}_{k}\right|^{5}}\right), \left|\vec{r}-\vec{r}_{k}\right|\geq p$. (3S)

Let us compare the analytical model dynamics of the magnetic moments of two clusters and the results of numerical modelling for two clusters (with immobile ions) to confirm the turning of the magnetic moments. The simulation tool we use is the 3D EPOCH particle-in-cell code. We assume an already-ionized spherical target (cluster). The ion charge state is = 20 and the ions are Xe ions with a number density of $n_{0}$= 10^22^ cm^−3^. The corresponding electron density in the target is 2 × 10^23^ cm^−3^,which is equivalent to $n_{e}=\text{ }182n_{cr}$in the case of $\lambda_{L}$= 1 μm radiation wavelength, where $n_{cr}$ is the critical density. The incoming laser pulse is a circularly polarized plane wave (the simulation domain is smaller than the laser focal spot size) with a Gaussian temporal field profile. The peak intensity of the laser pulse is $I_{L}$= 2 × 10^18^ W/cm^2^ and its duration is 6 fs. The simulation domain has a volume of 1.5 × 1 × 1 μm^3^ represented by 600 × 400 × 400 grid cells, and the target plasma is represented by 10 ion and 100 electron macroparticles per cell. The arrangement and the sizes of the clusters are shown in Fig. 1A.


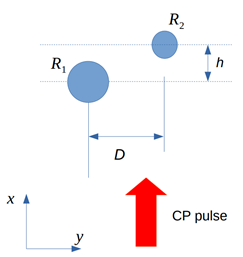


Fig. 1S Positions of two clusters in the modelling box. The x, y, z co-ordinates are given in nanometre. The circularly-polarized laser pulse falls in the positive direction of the x-axis. Both clusters have a z=0 co-ordinate.

We performed two simulations with two parameter sets: $D, h$ $D=100, h=40 nm$ (”large” distance between centres) and $D=50, h=0 nm$ ("small" distance between centres). Cluster electron trajectories for these two cases are shown in a Fig. 2S and 3S, respectively.

Fig.2S Electron trajectories of several sample electrons from t=0 to 60 fs. The co-ordinates are in microns. R_1_ =40 nm, R_2_ =40 nm, D =100 nm, h=40 nm, *I* = 2x10^18^ W/cm^2^, $\tau_{L}$=6 fs, Xe^+20^ cluster, *n_i_* = 3x10^22^ cm^-3^, 3D simulation box 1.5 × 1 × 1 μm^3^. Red colour – the first cluster electrons, dark blue colour – electrons from the second cluster.

Fig. 2S shows that electrons with a characteristic energy of $m_{e}c^{2}(\sqrt{1+a^{2}}-1)$ are localized around cluster ion cores and rotate in a YZ plane , and oscillate along the x-axis.

Fig.3S Electron trajectories of several sample electrons from t=0 to 60 fs. The co-ordinates are in microns. R_1_ =40 nm, R_2_ =40 nm, D =50 nm, h=0 nm, I = 2x10^18^ W/cm^2^, $\tau_{L}$=6 fs, Xe^+20^ cluster, *n_i_* = 3x10^22^ cm^-3^, 3D simulation box 1.5 × 1 × 1 μm^3^. Red colour – electrons of the first cluster, dark blue colour – the second cluster.

At a reduced distance between clusters, as apparent from Fig. 3S, an electron can transfer from the first cluster to the second one, and the electron clouds may merge. Clusters lose parameter similarity (charge and magnetic moment) as they are located at different "depths" in relation to the laser axis. Interaction of the electron clouds leads to effective collisionless friction force influencing the turn of the clusters’ magnetic moment. The comparison of the time dependence of the magnetic field components in the cluster centres ($\vec{H}_{1,2}(t)=2\mu_{1,2}\vec{n}_{1,2}(t)/p^{3}$, see Eq. (2S) with the parameters used in Fig. 1S) is presented in Fig. 4S. Numerical and analytical dependences coincide at $\mu_{2}\approx0.5\mu_{1}$, and the effective collisionless relaxation constant is $\tau_{cl}=300$ fs.

Fig. 4S Time dependence of the magnetic field components in the centre of each cluster. The left hand side shows the model (2A) results, while the right hand side – depicts the PIC-calculations for: R_1_ =40 nm, R_2_ =40 nm, D =50 nm, h=0 nm, I = 2x10^18^ W/cm^2^, $\tau_{L}$=6 fs, Xe^+20^ cluster, *n_i_* = 3x10^22^ cm^-3^, 3D simulation box: 1.5 × 1 × 1 μm^3^.

The comparison between the PIC simulation and the modelling results (2S, 3S) of the magnetic field’s z-components for the system of two dipoles a is presented in Fig. 5S.


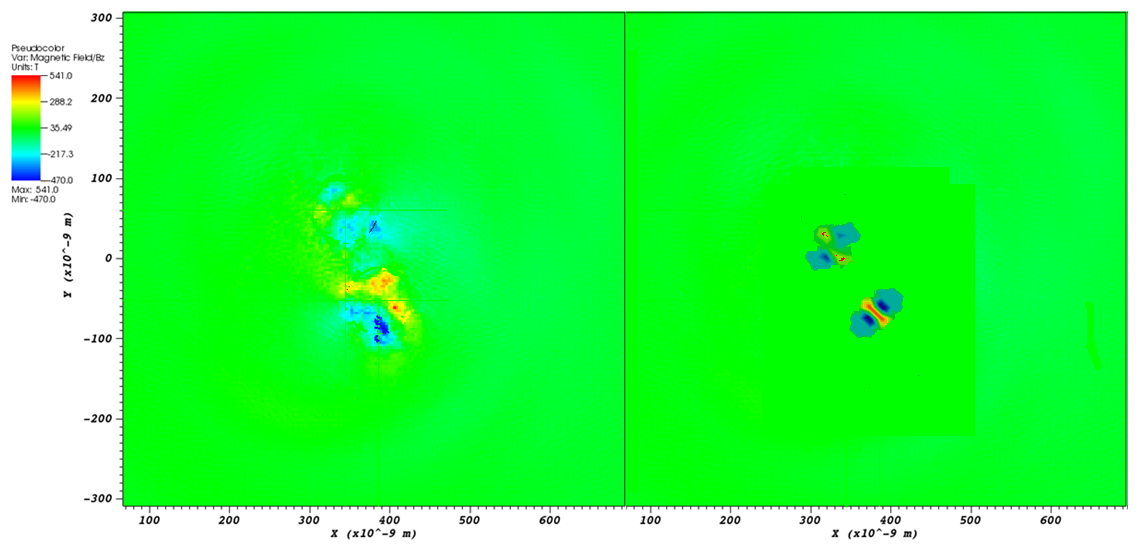


Fig. 5S Spatial distribution at time moment t=16 fs of z– magnetic field components (across the laser axis). The right hand side shows the model (3S) results for two dipoles, while the left hand side depicts the – PIC simulations for R_1_ =40 nm, R_2_ =40 nm, D =50 nm, h=0 nm, I = 2x10^18^ W/cm^2^, $\tau_{L}$=6 fs, Xe^+20^ cluster, *n_i_* = 3x10^22^ cm^-3^, 3D simulation box 1.5 × 1 × 1 μm^3^.

The right hand side of Fig. 5, shows the spatial distribution of the z-components of the magnetic field calculated from Eq. (3S), and it is in good agreement with the PIC simulations, shown on the left hand side. The configuration of the magnetic field of a separate dipole in the model possesses obvious axial symmetry. In the PIC simulations, due to the interaction of the electron clouds, the axial symmetry is lost and the configuration of the field is also more complicated. However, the conformity of the analytical model and the PIC simulation results is obvious from Fig. 4S and Fig. 5S.

**Magnonic oscillations in a four-cluster system with infinite ion mass**

We provided PIC-modelling of four Au^+30^ clusters of 100 nm radius irradiated by circularly polarized, 6 fs (super-Gaussian shape) laser pulse having an intensity of 5.6x10^20^ W/cm^2^. The box size was 3x3x3 μm^3^. The clusters were located at the peaks of a rhombus with 60º and 120 º angles and R_1_ = (510,-490, 0), R_2_ = (1490, 490, 0), R_3_ = (1000, 0, 400), R_4_ = (1000, 0,-400) nm, thus the distance between the nearest clusters was 800 nm. This cluster arrangement differs from Fig. 3 only in some factors. The result of the PIC simulations up to 400 fs is presented in Fig. 6S (see left hand side). It is visible that the magnetic moments of the clusters rotate around the x-axis, which means an approximately constant value for the field x-components, and the oscillations of the y and z components, with an establsihed phase shift of $\sim\pi/2$.

Fig.6S Components of the magnetic field of a four-cluster system with infinitely heavy ions, obtained from PIC simulations, (left) and from an analytical model for gold (right) with time of relaxation $\tau_{cl}\sim700$fs.

At picosecond relaxation times ($\tau_{cl}\sim700$fs), system (7) predicts a behaviour similar to the PICsimulations of the cluster’s y and z magnetic field components (and the magnetic moment), shown on a right hand side of Fig. 6S by lines having the same colours as those used in the PIC simulations. The x-components of the magnetic field in the simulations agree well with the model only up to ~150 fs. At later time instances, the oscillations continue in the model, but stop in the simulations and a constant value is established for the X-component. The possible reason behind the qualitative difference between numerical and model calculations of the dipoles can be partially associated with the electrostatic interaction of the clusters’ electron clouds in the simulation box limited by the available computing resources. More detailed research of the magnonic wave damping requires a larger simulation box and additional 3D calculations with a bigger distance between clusters. Such simulations are planned to be executed in subsequent works.
